# Improving morphological outcome in lamellar macular hole surgery by using highly concentrated autologous platelet-rich plasma

**DOI:** 10.1007/s00417-021-05486-5

**Published:** 2021-12-06

**Authors:** Felix Hagenau, Nikolaus Luft, Matthias Nobl, Denise Vogt, Julian E. Klaas, Benedikt Schworm, Jakob Siedlecki, Thomas C. Kreutzer, Siegfried G. Priglinger

**Affiliations:** University Eye Hospital, Ludwig-Maximilians-University, Mathildenstrasse 8, 80336 Munich, Germany

**Keywords:** Lamellar macular hole, LMH, Platelet-rich plasma, PRP, Peeling, Vitrectomy

## Abstract

**Purpose:**

To evaluate the use of highly concentrated autologous platelet-rich plasma (PRP) in lamellar macular hole (LMH) surgery with regard to function and morphology.

**Methods:**

We included 12 eyes of 12 patients with progressive LMH in this interventional case series. After 23/25-gauge pars plana vitrectomy, 0.1ml highly concentrated autologous platelet-rich plasma was applied under air tamponade. Induction of posterior vitreous detachment and peeling of tractive epiretinal membranes were performed whenever present. Phacovitrectomy was undertaken in cases of phakic lens status. Postoperatively, all patients were instructed to rest in a supine position for the first two postoperative hours. Best-corrected visual acuity (BCVA) testing, microperimetry, spectral-domain optical coherence tomography (SD-OCT), and fundus photography were carried out preoperatively and 6 months postoperatively.

**Results:**

Foveal configuration was restored in 10 of 12 patients (83.3%) at 6 months postoperatively. Two patients who had not undergone ILM peeling showed a recurring defect at 6-month follow-up. Best-corrected visual acuity improved significantly from 0.29 ± 0.08 to 0.14 ± 0.13 logMAR (Wilcoxon: *p*=0.028). Microperimetry remained unchanged (23.38 ± 2.53 preoperatively; 23.0 ± 2.49 dB postoperatively; *p*=0.67). No patient experienced vision loss after surgery, and no significant intra- or postoperative complications occurred.

**Conclusion:**

The application of PRP in the surgical therapy of LMH results in good morphological and functional outcomes. Additional peeling of the ILM seems to be mandatory when using PRP to prevent the recurrence of LMH. Strict postoperative supine positioning for 2 h avoids PRP dislocation. Larger sample sizes are needed to confirm the results.

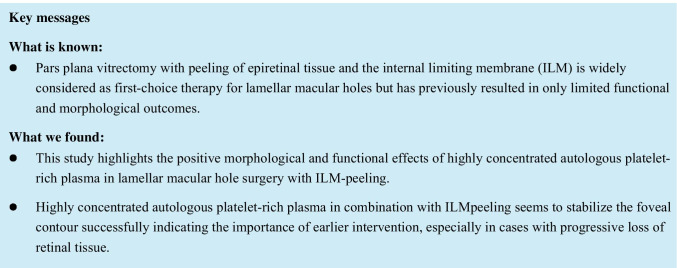



## Introduction

Lamellar macular holes (LMHs) belong to a spectrum of progressive diseases at the retinal and vitreoretinal interface. These entities sometimes exhibit distinct signs of degeneration of the retinal tissue and can therefore lead to decreased visual acuity and metamorphopsia. LMHs belong to the group of non-penetrating macular defects including pseudoholes and epiretinal membrane (ERM) foveoschisis; however, they differ, in particular, by the absence or markedly reduced appearance of tractive epiretinal tissue [1].

Diagnosis of LMH is based on spectral-domain optical coherence tomography (SD-OCT), wherein three mandatory criteria must be visible: an irregular foveal contour, a foveal cavity with undermined edges, and a sign of foveal tissue loss. Associated signs can be a so called foveal bump, the disruption of the ellipsoid zone, or epiretinal proliferation (ERP) [1]. These associated signs (especially the disruption of the outer retinal layers) indicate a longer duration of the disease and are related to reduced visual function [2, 3].

Although not included in the current OCT classification of non-penetrating macular defects, degenerative and tractive LMH can be distinguished within the group of LMH. These two LMH types show different behavior in terms of progression and surgical outcome [4].

Multimodal imaging has become a regular and central step in our decision-making process when a patient presents with symptoms and clinical findings of LMHs, because tractive epiretinal membranes can now be reliably assessed with regard to their morphological extent, reflectivity, and location [5].

The current standard of care for the treatment of LMHs is vitrectomy with epiretinal tissue and internal limiting membrane (ILM) removal. However, patient counseling with respect to surgical intervention is highly individual and is not only based on features found in OCT, but also on disease progression and subjective visual impairment. Moreover, previous studies have shown the reduced efficacy of vitrectomy in LMH, especially in the absence of additional tractive epiretinal components. Thus, recommendations for treatment are often delayed leading to disease progression and a possible poorer outcome after late therapy initiation. We therefore need safer and more effective therapeutic approaches in LMH.

Since the 1990s, platelet-rich plasma (PRP) has been described as an adjunct in the macular surgery of traumatic, persistent, and recurrent full-thickness macular holes or in optic disc pit maculopathy [6–10]. This method has not only been used in vitreoretinal surgery, but also in therapy for ocular surface diseases [11–14].

The main component of PRP is thrombocytes, which are also termed blood platelets. These cells are a natural reservoir for many growth factors that play a major role in wound healing, e.g., epidermal growth factor (EGF), nerve growth factor (NGF), platelet-derived growth factor (PDGF), transforming growth factor (TGF), basic fibroblast growth factor (bFGF), or vascular endothelial growth factor (VEGF) [15].

Recently, we have reported the 3-month outcome of highly concentrated autologous PRP in lamellar macular hole (LMH) surgery. We now present data from the 6-month follow-up [16].

## Materials and methods

This interventional case series included 12 eyes of 12 patients with symptomatic and progressive LMH. In all patients, vitrectomy was performed together with the application of highly concentrated autologous PRP. Surgery was carried out between December 2019 and November 2020 at the Department of Ophthalmology, Ludwig-Maximilians-University Munich, Germany, by two highly experienced vitreoretinal surgeons (SGP, TCK). This study was approved by the institutional review board of the University Eye Hospital of the Ludwig-Maximilians-University and was conducted in accordance with the tenets outlined in the Declaration of Helsinki. All subjects gave written informed consent before undergoing the interventions outlined below.

### Patient selection

All patients underwent clinical examination and multimodal imaging including SD-OCT. The diagnosis of LMH was confirmed when all three of the established mandatory SD-OCT criteria were fulfilled: (1) an irregular foveal contour, (2) a foveal cavity with undermined edges, and (3) signs of foveal tissue loss [1]. Confounding retinal pathologies such as diabetic retinopathy, vitreous hemorrhage, retinal detachment, age-related macular degeneration, inflammatory disease, vascular occlusion, high myopia <−6.00dpt, or trauma led to exclusion of the patient from the study, as did a history of vitreoretinal surgery. Recommendation for vitrectomy was based on a progression in morphology and/or a significant decrease in visual acuity during the preoperative follow-up period and/or significant impairment of quality of life caused by metamorphopsia.

### Preoperative and postoperative examinations

Best-corrected distance visual acuity (BCVA) was determined using standard ETDRS charts at 4 m, after subjective manifest refraction had been measured. Examination consisted of slit-lamp biomicroscopy including dilated fundus examination, SD-OCT scanning with volume and radial scans (SPECTRALIS® HRA + OCT, Heidelberg Engineering, Heidelberg, Germany), microperimetry (MAIA, Centervue Inc., Fremont, USA), and fundus photography (Optos P200Tx, Optos, Dunfermline, Scotland). Follow-up examinations took place at 1, 3, and 6 months postoperatively and included identical work-up.

### PRP preparation

The preparation of PRP followed the protocol described previously [16]. Whole-blood collected preoperatively (105ml) was anti-coagulated at a ratio of 1:7 and divided into its components by a special closed-circuit centrifugation procedure (Arthrex Angel System™; Arthrex, Naples, Florida, USA). In addition to platelet-poor plasma (PPP) and red blood cells (RBC), this method produced a highly concentrated PRP, which, because of the centrifugation mode, had a particularly low proportion of pro-inflammatory leukocytes compared with that obtained by the usual methods. The concentration of the platelets obtained was 8.8 times higher than that found in whole blood.

### Surgical procedure

All patients underwent vitrectomy with induction of PVD and peeling of epiretinal tissue depending on the extent of the tractional component as determined by the surgeon. The ILM was also peeled consistently, except in three cases.

The peeling of epiretinal tissue and ILM was performed after a staining step with MembraneBlue-Dual dye (0.125mg Brilliant Blue G and 0.75mg Trypan Blue, D.O.R.C., Zuidland, Netherlands) and at least a second control staining. All phakic patients underwent combined phacovitrectomy with implantation of a previously calculated intraocular lens.

Highly concentrated PRP (0.1ml, previously prepared) was then applied to the posterior pole under air tamponade. Following this procedure, all patients received air or gas tamponade. Postoperative rest in the supine position was strongly advised for the first two postoperative hours.

### Main outcome measures

The primary outcome measure was postoperative morphology on SD-OCT, such as the integrity of the inner and outer retinal layers and the inner foveal contour during all follow-up examinations. Secondary outcome measures were functional results, including visual acuity, microperimetry, and an assessment of metamorphopsia.

### Statistical analysis

Statistical analysis was performed using IBM® SPSS® Statistics Version 26 (IBM Corporation, New York, USA). Descriptive data are displayed as mean ± standard deviation (*SD*). The Wilcoxon signed-rank test was performed to compare two related samples (BCVA; data of microperimetry; central retinal thickness). A *p*-value of <0.05 was considered to indicate statistical significance.

## Results

Patient ages ranged between 57 and 81 years with a mean of 72 ± 8 years (median 74 years). The group consisted of seven male and five female patients. The main clinical data of all the patients at baseline and follow-up and their surgical characteristics are summarized in Table 1.

**Table 1:** Clinicial and surgical data

ID	Age/sex/eye	Lens status		VRI findings	Surgery	BCVA (logMAR)	
		Pre-op	Post-op		Peeling of structures	Pre-op	6 months FU
1	78/M/OD	IOL	IOL	LHEP; HET	ERM; ILM	0.4	0.1
2	81/M/OS	Phakic	IOL	LHEP	None	0.22	0.0
3	57/F/OD	Phakic	IOL	LHEP; HET	VC; ERM; ILM	0.22	0.22
4	67/F/OD	Phakic	IOL	LHEP; HET	ERM; ILM	0.22	-0.1
5	65/M/OS	IOL	IOL	LHEP; HET	ERM; ILM	0.4	0.3
6	71/M/OS	Phakic	IOL	LHEP; HET	VC	0.3	0.22
7	61/F/OD	Phakic	IOL	HET	VC; ERM; ILM	0.22	0.1
8	80/M/OD	IOL	IOL	LHEP; HET	VC; ILM	0.22	0.22
9	76/F/OS	IOL	IOL	LHEP	ILM	0.3	0.3
10	71/M/OD	IOL	IOL	LHEP; HET	ERM; ILM	0.22	0.22
11	78/F/OD	Phakic	IOL	LHEP	ILM	0.4	0.4
12^a^	79/M/OD	IOL	IOL	LHEP; HET	VC; ILM (2nd surgery)	0.4	0.0

*M*, male; *F*, female; *OD*, right eye; *OS*, left eye; *IOL*, intraocular lens; *LHEP*, lamellar hole–associated epiretinal proliferation; *HET*, hyperreflective epiretinal tissue; *ERM*, epiretinal membrane; *ILM*, internal limiting membrane; *VC*, vitreous cortex; *BCVA*, best-corrected visual acuity; *VA*, visual acuity; *FU*, follow-up; ^a^patient underwent re-vitrectomy with ILM peeling due to dislocation of PRP

Lens status was evenly distributed (six phakic and six pseudophakic eyes). All six phakic eyes underwent vitrectomy combined with phacoemulsification and implantation of an intraocular lens.

### Functional outcomes

Preoperatively, the mean best-corrected visual acuity (BCVA) was 0.29 ± 0.08 logMAR (median 0.26; range 0.4 to 0.22) corresponding to 20/40 Snellen. The mean BCVA of the entire cohort increased at 6 months postoperatively to 0.14 ± 0.13 logMAR (median 0.14; range 0.3 to −0.1) corresponding to 20/25 Snellen and resulting in a significant VA improvement (*p* = 0.028, Wilcoxon test). The mean BCVA of the pseudophakic cohort at baseline was 0.32 ± 0.09 logMAR (median 0.35; range 0.4–0.22) and 0.19 ± 0.12 logMAR (median 0.22; range 0.30–0.00) at 6 months postoperatively resulting in a non-significant VA improvement (*p*=0.109). No patient showed a loss of lines after intervention.

Microperimetry showed a preoperative mean threshold of 23.38 ± 2.53 dB (median 23.8, range 18.5–26.8). At 6 months postoperatively, the threshold remained stable at 23.0 ± 2.49 dB (median 23.4; range 18.8–26.10; *p* = 0.67 Wilcoxon test). Fixation stability values P1 and P2 also showed no significant change from a P1 median of 75 ± 29.5% preoperatively to 84 ± 30.5% at the 6-month follow-up (*p* = 0.9, Wilcoxon test) and a P2 median of 93 ± 15% preoperatively to 96 ± 16.3% at the same follow-up (*p* = 0.4, Wilcoxon test).

### Morphological outcomes

Preoperatively, all patients fulfilled the three mandatory criteria for LMH on SD-OCT, presenting with an irregular foveal contour, a foveal cavity with undermined edges, and signs of foveal tissue loss.

Postoperatively at 3 months, the foveal contour showed an improved configuration in all cases (12 of 12) without signs of tissue loss after the endotamponade was resorbed.

**Fig. 1:**
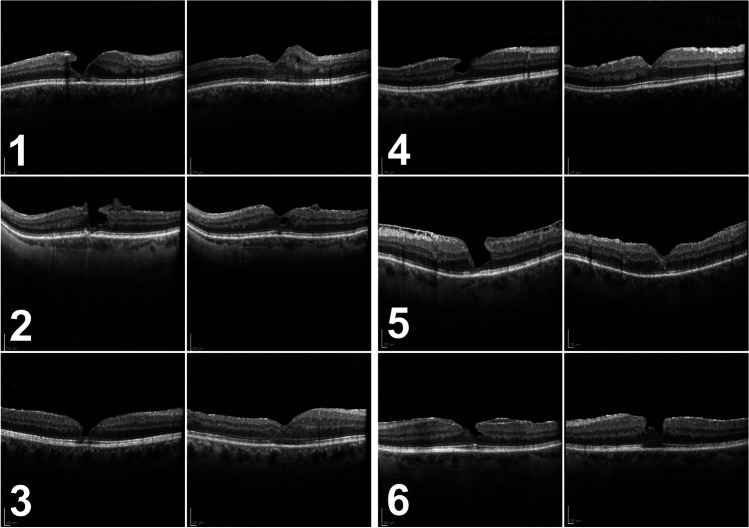
Preoperative (left) and 6-month postoperative (right) spectral-domain optical coherence tomography of patients #1–6

**Fig. 2:**
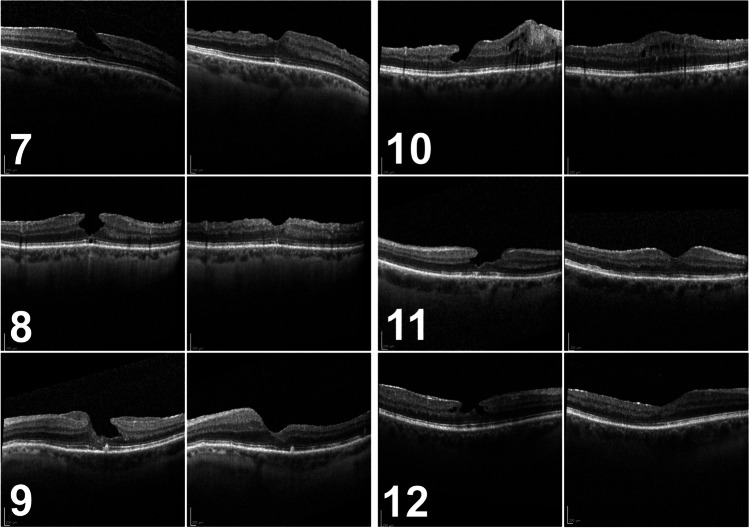
Preoperative (left) and 6-month postoperative (right) spectral-domain optical coherence tomography of patients #7–12

After 6 months, we observed a recurrence of the tissue defect in two patients (Fig. 1: 2 and 6), namely, in two of the three patients who had not received ILM peeling during initial surgery. The other 10 cases showed stable foveal morphology at month 6 (Fig. 1 and Fig. 2).

**Fig. 3:**
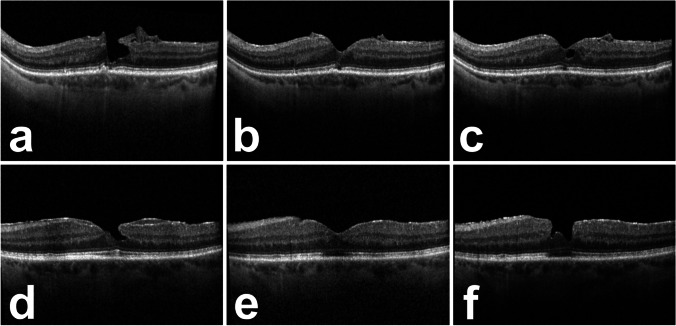
Spectral-domain optical coherence tomography of patient #2 and #6 at baseline (**a**/**d**), at 3 months (**b**/**e**), and at 6 months showing a recurrent foveal tissue defect (**c**/**f**)

The recurrence of the tissue defect was not evident until the follow-up at month 6 after a good morphological result at 3 months. (Fig. 3) The defect led to an increase of metamorphopsia in one of two patients, although visual acuity remained stable at the 6-month follow-up in both patients.

**Fig. 4:**
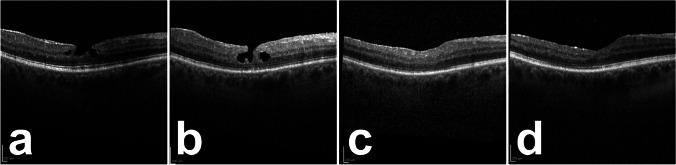
Spectral-domain optical coherence tomography of patient #12 at baseline (**a**), at 1-week follow-up after postoperative PRP dislocation with persistent lamellar defect (**b**), at 3-month follow-up after second vitrectomy (**c**), and at 6-month follow-up (**d**)

The third patient who had not received ILM peeling had not adhered to the strict postoperative supine positioning for 2 h. In this case, immediate dislocation of the platelet plug was observed at one day after surgery and was confirmed on follow-up SD-OCT at 1 week postoperatively. (Fig. 4a–b) After the SF6-endotamponade had dissolved, a second vitrectomy with ILM peeling and repeated addition of PRP was performed. Closure of the LMH with improved foveal contour was observed in this patient at months 3 and 6, together with excellent functional improvement (Fig. 4c–d).

Postoperative cystoid edema was present in 3 of 12 eyes (25%) and was treated with non-steroidal anti-inflammatory eye drops (Nepafenac 0.3%) or parabulbar steroid injection, if persistent.

The central macular thickness (CMT) in the 1mm circle of an overlying ETDRS-grid increased from 291.42 ± 30.53 μm (range 240-348μm) preoperatively to 329.2 ± 51.46 μm (277–425) at the 3-month follow-up (*p*=0.017, Wilcoxon test).

## Discussion

In the present study, we show that the additional use of PRP applied to the fovea after standardized vitrectomy and ILM peeling improves macular anatomy and retinal function. These results might change clinical practice, since patients with LMH are, at present, most likely to be recommended close monitoring and no surgical intervention. The degenerative properties of LMH together with the results obtained in previous studies seem to have led to the perception that, contrary to tractional vitreoretinal diseases, only limited success is achieved by a surgical approach in LMH [17]. However, more recent studies have suggested the possible advantages of earlier surgical intervention, even in the absence of any tractional force on SD-OCT [18]. A recent meta-analysis has shown no differences in improvement, irrespective of whether tractive elements are present [19].

LMHs tend to progress in at least one-third of patients and can lead to disruption in the ellipsoid zone [20]. This is associated with a worse visual outcome, even after interventional treatment by vitrectomy [21]. Chehaibou et al. have shown that, when further complications occur, such as an associated full-thickness macular hole (FTMH), then these cases tend to have poorer surgical outcomes when compared with those with idiopathic FMTH [22].

Based on the current safety profile of minimally invasive vitrectomy together with the meta-analyses regarding visual outcome, early intervention might be considered in order to stabilize the foveal structures and to prevent potential future progression. Of course, severe complications of an interventional approach, such as progression to FTMH or retinal detachment, have to be taken into account in the decision-making process and carefully explained to the patient [20].

However, some limitations of our study should also be mentioned here. We cannot clearly state whether the functional improvement that we have observed, but that was not seen in previous larger studies involving the surgical management of LMH, is attributable to the diagnostic criteria, the patient population, or the surgical method [21, 23]. More recent studies with also smaller case numbers showed similar functional results as we did [19, 24, 25]. An important point to consider in comparisons of studies of LMH is the heterogeneity in diagnosis. This is now becoming much more uniform with the help of the SD-OCT classification. The inhomogeneity of the cases with respect to the preoperative lens status is also problematic, since subsequent phacovitrectomy is performed in cases of preoperative phakic lens status. Furthermore, we have only studied a small number of cases, with no control group. Whereas our study has yielded promising results, we hope that future randomized controlled trials with larger cohorts will refine the evidence and confirm recommendations for the regular use of highly concentrated PRP in LMH surgery.

Our research indicates that the use of PRP should be accompanied by the mandatory peeling of the ILM. In comparison, vitrectomy with the induction of posterior vitreous detachment (PVD) and an additional PRP application without ILM peeling does not seem to lead to the beneficial properties of PRP, whereas our 3-month results have revealed closure of the foveal defect, independent of ILM peeling.

These observations are consistent with the postulated mechanism of highly concentrated preparations of autologous PRP. The hypothesis behind the action of PRP is the activation of Mueller cells, the most frequent type of glial cells in the retina. Although definitive roles still cannot be assigned to Mueller cells, they are thought to have an important function in retinal physiology and structure. PRP is mainly composed of platelets, which are activated by contact with disintegrated neuroretinal tissue. Activation leads to the release of their growth factors, which are in turn able to stimulate Mueller cells and their proliferative and migrating properties [26]. We therefore propose that persistent ILM after surgery is a barrier with regards to platelet activation and thus recommend ILM peeling in LMH surgery, especially if combined with autologous PRP.

One learning point to be drawn from this study, in addition to the effect of ILM peeling, is the importance of the strict supine positioning of the patient, after surgery, in order to prevent the dislocation of foveal PRP. In one patient, only a brief period in an upright position in the immediate postoperative period caused the platelet concentrate to slip away from the macula. Patients must therefore strictly maintain a supine position for at least 1.5h or, even better, 2h after surgery.

Based on the unequivocal anatomical results from SD-OCT, our study clearly indicates a positive effect of the additional application of the highly concentrated PRP in vitrectomy for LMH. The anatomical reconstruction of the foveolar contour of LMHs, especially in cases with only small tractive forces, was previously unimaginable. Our morphological and functional results may thus open the way for earlier intervention, in particular for degenerative LMHs; this would truly represent a paradigm shift in the treatment of LMH.

Previously published data have established that LMH can deteriorate continuously, if left untreated [20]. Thus, we consider that the demonstrated improvement in the morphological structure, together with the stabilization of visual acuity, can be considered as a success for patients suffering from LMH and its associated spectrum of clinical symptoms. Further studies with a larger number of cases and longer follow-up times are needed to determine the duration of the functional and morphological benefits of PRP after successful vitreoretinal surgery.

## Declarations

### Data availability

Relevant documentation or data in order to verify the validity of the results can be sent upon request.
